# Undertaking a New Regulatory Challenge: Monitoring of Ergot Alkaloids in Italian Food Commodities

**DOI:** 10.3390/toxins13120871

**Published:** 2021-12-06

**Authors:** Veronica Maria Teresa Lattanzio, Emanuela Verdini, Stefano Sdogati, Angela Caporali, Biancamaria Ciasca, Ivan Pecorelli

**Affiliations:** 1 National Research Council of Italy, Institute of Sciences of Food Production, Via Amendola 122/O, 70126 Bari, Italy; veronica.lattanzio@ispa.cnr.it (V.M.T.L.); biancamaria.ciasca@ispa.cnr.it (B.C.); 2 Chemistry Department, Istituto Zooprofilattico Sperimentale dell’Umbria e delle Marche “Togo Rosati”, 06126 Perugia, Italy; e.verdini@izsum.it (E.V.); stefano.sdogati@izsum.it (S.S.); a.caporali@izsum.it (A.C.)

**Keywords:** ergot alkaloids, LC-MS/MS method, wheat, cereal products, occurrence

## Abstract

The present manuscript reports on monitoring data of 12 ergot alkaloids (EAs) in cereal and cereal-derived products, collected in Italy over the period 2017–2020, for official control purposes under the edge of the Commission Recommendation 2012/154/EU on the monitoring of the presence of EAs in feed and food. To these purposes, an LC-MS/MS method was set up and applied, after in-house verification of its analytical performance. Besides satisfactory recoveries and precision, the method’s quantification limits proved suitable to assess the compliance of cereals and cereal-based foods with the recently issued EU maximum permitted levels (Commission Regulation 2021/1399/EU). The validity of the generated data was also evaluated through the adoption of four proficiency tests, from which acceptable z-score values (−2 ≤ z ≤ 2) were obtained. The method was then applied to analyse a total of 67 samples, collected in Italy over the period 2017–2020. The samples consisted of 18 cereal grains, 16 flours (14 of wheat and 2 of spelt) and 31 other types of cereals derivatives (including 9 for infants). Overall, the EAs analysis returned a high percentage of left-censored data (>86%). Among the positive samples, the highest contamination levels, up to 94.2 µg/kg, were found for ergocristine (12% incidence), followed by ergocristinine (7% incidence) with levels of up to 48.3 µg/kg.

## 1. Introduction

The EAs are mycotoxins produced by several species of fungi in the genus *Claviceps*. In Europe *Claviceps purpurea* is the most widespread and it commonly affects cereals such as rye, wheat, triticale, barley, millets and oats [1]. During fungi infection, healthy kernels are replaced by dark mycelial masses known as sclerotia (also known as ergots, or ergot bodies) that contain high concentrations of various EAs [2].

The toxicity of EAs is well known and has been characterized [3,4]. Though some are cytotoxic and antimicrobial, most are primarily neurotropic. Today, ergotism has practically been eliminated as a human disease, but it remains an important veterinary problem, particularly in cattle, horses, sheep, pigs and poultry [5].

**Figure 1:**
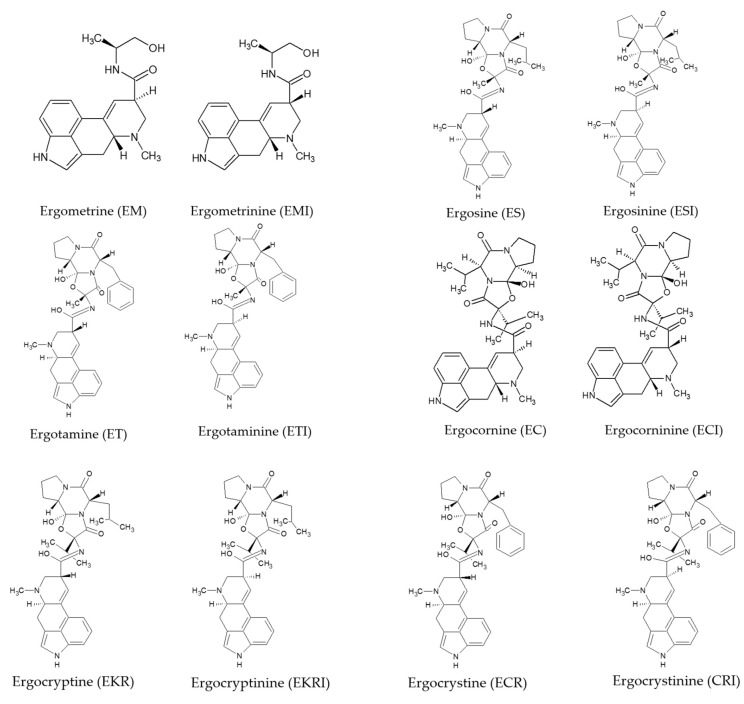
Structure of ergot alkaloids.

Based on the twelve EAs predominantly present in the sclerotia of *C. purpurea*, the EFSA Panel on Contaminants in the Food Chain (CONTAM Panel) concluded that chemical analysis should focus mainly on ergometrine (EM), ergometrinine (EMI), ergosine (ES), ergosinine (ESI), ergotamine (ET), ergotaminine (ETI), ergocornine (EC), ergocorninine (ECI), mixture of α- and β-isomers of ergocryptine (EKR) and ergocryptinine (EKRI), ergocristine (ECR) and ergocristinine (ECRI) (Figure 1). The -inine epimers are described to be biologically inactive, however, an interconversion occurs under alkaline or acidic conditions and, thus, the CONTAM Panel based its risk assessment on both forms (-ine and -inine) [3].

Although, today, advanced cleaning procedures prior to milling are rather effective, EAs are still found in food and feed commodities, sometimes at relatively high levels [6,7]. The occurrence data on EAs in food and feed submitted to EFSA indicates that ET, ECR, ES and EC mostly contribute to the overall content of EAs. Furthermore, the highest concentrations of EAs were reported for rye (grains, milling) products and by-products [3,4]. The CONTAM Panel recommended, that efforts should continue to collect more data on occurrence of the above EAs in relevant food and feed commodities. Special attention should be paid to processed food and to specific foods consumed by vegetarians or raw grain consumers. Moreover, the CONTAM Panel underlined the need for commercially available reference standards, such as for isotope-labelled internal standards and certified reference materials (CRM) for the analysis of EAs.

As a follow up to the conclusions and the information contained in the EFSA opinion, a Commission Recommendation on the monitoring of the presence of EAs in feed and food has been in force since 2012 to stimulate analytical data collection regarding the occurrence of EAs identified in the EFSA opinion in relevant food and feed commodities. Furthermore, the Commission Recommendation encouraged the collection of specific information on the relationship between the presence of ergot sclerotia and the level of individual EAs in food and feed in order to set appropriate limits [8]. Finally, in response to EFSA Recommendation, regarding “harmonised performance criteria for the analysis of EAs in feed and food” [3], the Committee agreed that the method of analysis used for the monitoring of ergot alkaloids should have a limit of quantification (LOQ) of 20 μg/kg as a minimum acceptable criterion, but preferably, this value should be 10 μg/kg or lower [9].

Recently, on 24 August 2021, the European Commission published Regulation (EU) 2021/1399 [10], amending Regulation (EC) 1881/2006 [11]. The new Regulation sets the maximum permitted limits for the sum of the above mentioned 12 EAs as lower-bound concentrations (i.e., calculated on the assumption that all values of the different individual ergot alkaloids below the limit of quantification are equal to zero) in cereal-based food products (Table 1). The limits for these alkaloids relate to barley, wheat, spelt, oats, rye and processed cereal-based foods for infants and children and will apply from 1 January 2022. In the Regulation, a higher maximum permitted level is set for milling products containing bran (identified on the base of ash content) taking into account the absorption by cereals of dust containing high levels of EAs.

The maximum levels of EAs set in Regulation (EU) 2021/1399 imply that the analytical methods, for enforcement purposes, should have a LOQ lower than the value previously established by the EU document [9]. Specifically, if calculated according to the formula reported in the UNI CEN/TR 16059:2010 [12] the LOQ for monitoring of milling products, bran milling products/grain for human consumption for cereal other than rye and processed cereal-based baby foods shall be equal to 4.0, 6.0 and 0.8 µg/kg per each ergot compound, respectively.

Different methods have been reported in the literature for the analysis of ergot alkaloids, mainly liquid chromatographic methods coupled to fluorometric or tandem mass-spectrometric detectors (FLD or MS/MS). A critical review can be found in Chung 2021 [13], discussing the advantages and disadvantages of available methods for determination of EAs in cereals and feed, covering the period from 2008 to 2020. The review points out that, although both LC-FLD and LC-MS/MS can be used for the analysis of the 12 EU-recommended EAs, the latter has a greater sensitivity, but it is affected by a matrix effect especially, for EM and EMI. Another analytical challenge, stressed in the review, is the co-elution of alpha and beta isomers of EKR (α-EKR, β-EKR) for most of the reported methods, due to the use of C18 analytical columns. A proficiency test, conducted in 2017, revealed that an acceptable resolution was obtained with phenyl-hexyl as a stationary phase [14]. Finally, the review underlines that only very few reported methods can fulfil the regulated LOQs for individual epimers in processed cereal-based food for infants and young children owing to its lower limit. Recently a modified QuEChERS-based method coupled to LC-MS/MS as a detection technique was successfully validated for the detection and quantification of EAs in dry cereal-based baby foods with individual LOQs of 0.5 µg/kg [15], however the method did not provide the separation of the α and β isomers of EKR and EKRI.

A standardized method, for the determination of EAs in cereals and cereal products by dispersive solid phase extraction (dSPE) clean-up and LC-MS/MS, has been recently issued by the European Committee for Standardization for official control purposes [16]. The method has been validated in the range of 13.2 µg/kg to 168 µg/kg for the sum of the twelve EAs, in rye flour, rye bread and cereal products (breakfast cereals, infant breakfast cereals and crispbread) that contained rye as an ingredient, as well as seeded wholemeal flour and a barley and rye flour mixture. Method performances were satisfactory in the range 24.1 µg/kg to 168 µg/kg for sum of EAs, whereas for concentrations below 24.1 µg/kg the method resulted to be only suitable for screening purposes.

Due to analytical challenges in the EAs determination, the occurrence of data available in the literature are scarce and provide a limited picture of EAs distribution worldwide.

The present manuscript reports on monitoring data of EAs in cereal and cereal-derived products collected in Italy over the period 2017–2020, as requested by the national implementation of the monitoring recommendations [8]. To these purposes a LC-MS/MS method for the determination of EAs in cereal and derived products has been optimized and in house validated to verify its fitness for purpose. Validation data will be reported and discussed, also taking into account the recently issued Regulation requirements.

## 2. Results and Discussion

### 2.1. Method Set Up and In-House Validation

The aim of this work was to set up and validate a fit-for-purpose method for the routine monitoring of EA in official control. Since the CEN standard [16] was not yet available at the time of the study, a new method was set up, starting from the procedure developed by Kokkonen et al. 2010 (https://doi.org/10.1002/jssc.201000114, accessed on November 2021). The primary improvements adopted to make the method suitable for routine analysis were a shorter extraction time (shaker time of 30 min vs. 60 min) and the use of a calibration curve, in the mobile phase, instead of a matrix-assisted calibration curve for quantification. This last point was very important for official controls, considering that different types of food products are generally analysed in the same batch.

Prior to the validation study, the chromatographic separation of target EAs, was optimized. Special attention was paid to EKR and EKRI, which have been shown to be particularly challenging under conventional reverse-phase chromatographic conditions, leading to chromatographically unresolved double peaks for both compounds, corresponding to the α- and β-forms [14].

**Figure 2:**
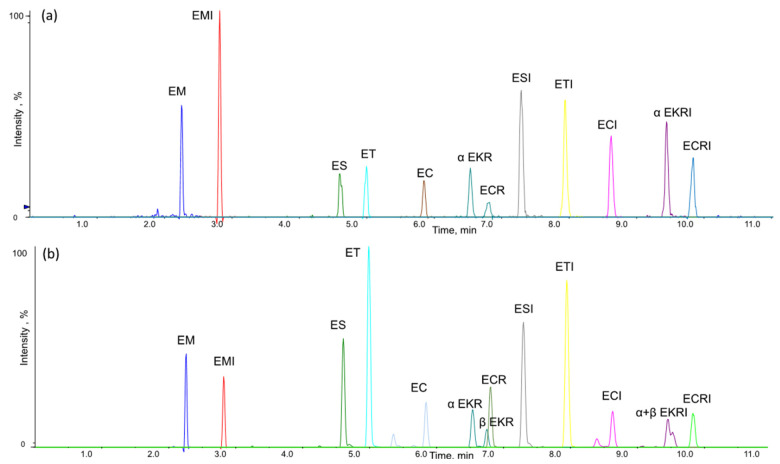
LC-MS/MS chromatogram of (**a**) wheat sample spiked with 2.5 µg/kg of each EA (EKR and EKRI alpha isomer only) and (**b**) naturally contaminated barley sample with EM (127 µg/kg), EMI (50 µg/kg), ESI (197 µg/kg), ET (858 µg/kg), ETI (209 µg/kg), EC (266 µg/kg), ECI (141 µg/kg), sum of α + β EKR (262 µg/kg), sum of α + β EKRI (119 µg/kg), ECR (459 µg/kg) and ECRI (161 µg/kg).

Within this study, two different reverse-phase columns were selected and tested to improve EKR and EKRI separation: a Kinetex EVO C18 (100 × 2.1 mm, i.d. 2.6 μm) and an Acquity UPLC BEH C-18 (150 × 2.1 mm, i.d. 1.7 μm). Complete separation of 12 EAs was achieved using column Acquity UPLC BEH C-18 as reported in Figure 2.

**Figure 3:**
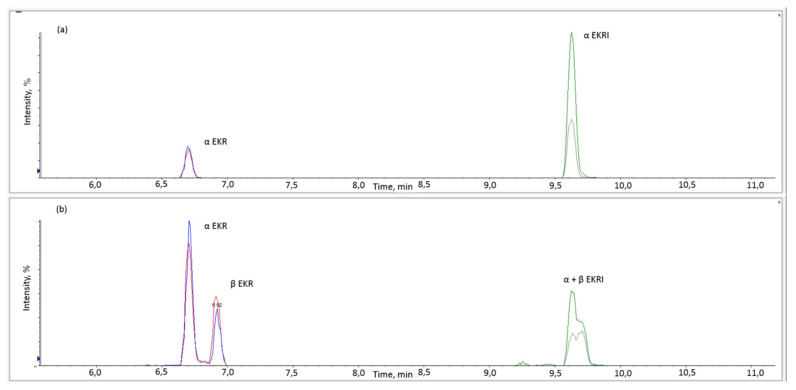
Extracted ion chromatograms (quantifier and qualifier transitions) for EKR and EKRI in standard solution (0.04 µg/mL) (**a**) and wheat sample naturally contaminated with EKR (mixture of α-EKR and β-EKR) (351 µg/kg) and EKRI (141 µg/kg) analysed for proficiency test using an Aquity BEH C-18 column (**b**).

Both columns were able to separate α and β isomers of EKR, while for EKRI, the Kinetex column did not provide any separation. For this reason, the Acquity UPLC BEH C-18 column was chosen. The EKR and EKRI results are shown in Figure 3.

Although the separation of the a- and β-isomers, co-occurring in real samples, would be desirable, a joint quantification (estimating the sum expressed as α isomer) might still be acceptable, in routine monitoring, considering the lack of available reference standards for the β forms.

Given that EAs are more likely occurring in cereals and relevant derived products, the in-house method’s performance was evaluated with wheat at concentrations as low as possible (e.g., the estimated LOQ) and at higher levels, taking into account available occurrence data.

Data obtained from in-house validation with wheat are summarized in Table 2.

Taking into account that no acceptability criteria for linearity were set in the EU legislation regarding performance criteria for mycotoxins analysis, the authors used residuals to evaluate linearity, and the criteria was met for all 12 compounds [17].

The estimated LOQs (see Section 4.6.1) ranged from 0.6 to 2.3 µg/kg for each compound and were compliant with CEN TR 16059 criteria. According to this guideline, when the legal maximum limit (ML) is set for a sum, the LOQs suitable for enforcement of the legal limit shall be equal to or less than ML divided by 2n (where n is the number of compounds involved). Therefore, the desired values for the monitoring of wheat-milling products and bran-milling products/grain for human consumption (other than rye) shall be set at 4.0 and 6.0 µg/kg for each ergot compound respectively. The values calculated according to the Guide are above the LOQs obtained for the present method.

Then, taking into account the experimentally determined LOQs values, the lowest validation level was set at 2.5 μg/kg for each individual toxin, whereas the others were set at 2 and 5 × LOQ. Mean recoveries ranged from 87 to 119%, whereas repeatability (RSD_r_) and within-laboratory reproducibility (RSD_WLR_) were lower than 13% and 15%, respectively (Table 2). Overall, very satisfactory performances were obtained for the proposed method.

A further confirmation of the reliability of the results obtained with the validated method should be sought in the positive outcome of the participation in four Proficiency Tests (FAPAS 22158, Rye Flour 2019, individual EA range 3–65 µg/kg; Bipea 99-1 Barley 2020, individual EA range 50–858 µg/kg; Bipea 99-2 Rye 2020, individual EA range 18–329 µg/kg; Bipea 99-3 Wheat 2020, individual EA range 76–1030 µg/kg).

Acceptable z-score values (−2 ≤ z ≤ 2) were obtained in all PTs (for a total of *n* = 49 provided results), even in cases where the values of the individual molecules were close to or even slightly lower than the estimated method LOQs.

### 2.2. Applicability of the New Method for Official Control Purposes

To provide evidence of the applicability and fitness for purpose of the presented method for official controls, data generated within the Italian national monitoring program on the period 2017–2020 are reported herein. Occurrence data for EAs are summarized in Table 3, whereas individual data for each toxin in all analysed samples are provided as Supplementary Material (Table S1).

The analysis of the EAs returned a high percentage of left-censored data (>86%). EM was the most abundant compound, followed by ECR and ES. The individual highest concentration was detected for ECR at 94.2 µg/kg in wheat bran. One sample only (wheat bran) contained all 12 EAs, with a sum of EAs of 271 µg/kg, which could be labelled as non-compliant under the new EU ML of 150 µg/kg [10]. All the other 16 positive samples were compliant, according to the relevant EU ML.

The data in Table 3 were then compared with previously generated ones. The most recent occurrence data for food samples, available in EFSA reports, cover the period 2011 and 2016 and show the highest average contributors to the total concentration to be ET (18%), ECR (15%) ES (12%) and EM (11%) [4]. Considering the large amount of left-censored data, present in the EFSA data set (86%), to minimize the impact of presence of relatively high LODs/LOQs on the UB (upper bound) scenario, a value of 20 µg/kg was selected as a LOQ cut off for each individual EAs, permitting the exclusion of those samples analysed by methods with poor sensitivity but without excessively compromising the number of available samples. In this respect, the LOQs of the method validated and applied in this manuscript were around 10 times lower than the above cut off level (Table 1) and, for this reason, can be considered fit for the purpose of an accurate occurrence evaluation.

The method was also applied to detect the presence of 12 EAs in cereal products for infants. The method did not report any particular issue; therefore, a future validation in cereal products for infants could be demonstrate its suitability for these product categories.

Available literature data on EAs occurrence in food samples, collected in the period 2015–2021, are summarized in Table 4. Results presented in this work are globally in line with previous studies. EM was also reported as the most common EA in wheat sample from Italy by Debegnac et al. [18], moreover, ECR was predominant in cereal samples from Luxembourg [19] and in French cereals [20].

The literature data provide a limited picture of EAs distribution worldwide. This could be partly attributed to the analytical challenges to be undertaken in analysing EAs. Therefore, the availability of isotopically internal standards could improve the accuracy of quantification. Moreover, the difficult chromatographic separation of alpha and beta EKR and EKRI isomers [14], the carefulness needed in samples and standard management (to avoid the epimerization of EAs during sample treatment) [13,21], make the analysis of EAs very tricky. The highest EA incidence is reported for rye and rye-based products, whereas an incidence lower than 10% was observed for other cereals and derived products, and, therefore, comparable to the data presented herein.

Overall, this comparison demonstrated that general applicability of the proposed method and, specifically, that (i) the ranges selected for method’s validation encompassed the natural contamination of EAs, not only in Italy, but also in other countries; and (ii) method quantification limits are also suitable to assess EAs contamination in samples for other countries.

The proposed method was suitable to monitor the natural occurrence of EAs in grain and cereal and derived products. Although the method was not validated on cereal products intended for infant consumption, it was applied to the analysis of nine cereal-based food for infants. From the results obtained, the method seems compliant, however further efforts are needed to lower the LOQ.

## 3. Conclusions

A fit-for-purpose LC-MS/MS method has been developed and validated for the determination of EAs in official control. The method’s performances were proven to be suitable in assessing the compliance of cereals and cereal-based foods with the recently issued EU maximum permitted levels (Commission Regulation 2021/1399/EU). Furthermore, the method’s applicability was evaluated by implementing it for EAs analysis in the national monitoring program, which included a total of 67 cereal-based samples collected in Italy over the period 2017–2020. Both the generated data and a comparison with previously reported occurrence data indicate that the method’s performances, in terms of precision, accuracy, applicability range and quantification limits, are suitable for assessing EAs natural contamination of cereals and derived products.

## 4. Materials and Methods

### 4.1. Chemicals and Reagents

EAs were obtained from Romer Labs (Tulln, Austria). Acetic acid and ammonium carbonate were purchased from Honeywell (Wunstorferstrasse, Germany). Ethyl acetate (EtOAc), Methanol (MeOH) and Acetonitrile (ACN) were obtained from Carlo Erba reagent Srl (Milan, Italy). All solvents used were of LC–MS or analytical grade. Water was purified by a Milli-Q system (Millipore, Merck KgaA, Darmstadt, Germany). The MycoSep 150 Ergot columns were purchased from Romer Labs.

### 4.2. Samples

Sixty-seven official samples were collected in the period between 2017 and 2020 from three six Italian Regions (Umbria, Marche and Puglia) and analyzed by Istituto Zooprofilattico Sperimentale of Umbria and Marche “Togo Rosati”. The samples consisted of 18 cereal grains, 16 flours (14 of wheat and 2 of spelt) and 33 other types of cereals derivatives (including 9 for infants) respectively.

Samples were ground by a knife mill (GRINDOMIX GM 300, Restek, Haan, Germany) with dry ice and split in aliquots of 25 g for the analysis. The samples were stored at −20 °C until analysis.

### 4.3. Reference Materials and Working Solutions

All reference materials (RMs) of EAs were in desiccated form. Reference solutions were prepared by reconstitution, according to the manufacturer’s instructions, obtaining a final concentration of 100 µg mL^−1^ for the R epimers of the EAs (ine-epimers) and 25 µg mL^−1^ for S epimers of EAs (inine-epimers) respectively. The obtained RMs solutions were stored in amber vials at −20 °C.

The working solutions (WS) were prepared by dilution of RMs just before use. For EAs-ine epimers an intermediate working mixed solution at 5 µg mL^−1^ was prepared. The intermediate solution of -ine epimers was then combined with single RMs of EAs-inine epimers to obtain a final concentration of 0.5 µg mL^−1^ for each molecule.

### 4.4. Sample Preparation

Twenty-five grams of sample were weighed in a 250-mL plastic vessel and 100 mL of extraction solution of acetonitrile:ammonium carbonate (200 mg L^−1^ (84:16 *v*/*v*)) were added. The samples were mechanically shaken for 30 min. After 15 min of centrifugation at 2780 RCF, 5 mL of extract was collected and loaded into the solid-phase extraction column (MycoSep 150 Ergot). One mL of purified extract was evaporated to dryness at 60 °C under a gentle stream of nitrogen. Finally, the sample was reconstituted with 400 µL of ammonium carbonate solution (200 mg/L)/ACN; (50:50 *v*/*v*) and filtered using a 0.2-μm PTFE syringe filter prior to injection into the LC-MS/MS system.

### 4.5. LC-MS/MS Analysis

The LC-MS/MS instrumental set up consisted of a Nexera X2 UPLC system (LC-30AD binary pump, CTO-20AC column oven and SIL-30AC autosampler, Shimadzu, Kyoto, Japan, 2015) interfaced to an API 3200 Qtrap mass spectrometer (AB Sciex, Foster City, CA, USA, 2009) equipped with an electrospray (ESI) ion source.

The analysis of EAs was performed in positive ionization mode (ESI+), after separation on an Acquity UPLC BEH C-18 (150 × 2.1 mm, i.d. 1.7 μm) connected to a VanGuard (2.1 × 5 mm) both from Waters (Milford, MA, USA). The column oven was set at 40 °C. The flow rate of the mobile phase was 500 μL/min, while the injection volume was 5 μL. Eluent A was a 200-mg/L ammonium carbonate solution and eluent B was acetonitrile. For EAs elution, the starting composition of the eluent was 95% (A) and 5% (B). Then, the following gradient was used: the proportion of eluent B was linearly increased from 5% to 40% over 1 min, then to 50% over the next 3.5 min, then increased to 70%. Finally, it was raised to 99% over 1.5 min and kept constant at 5% for 3 min.

The target mycotoxins were detected in Selected Reaction Monitoring (SRM) mode. The monitored transitions and retention times of single EAs are provided in Table 5. Compliance with SANTE mycotoxin identification criteria for retention time (Rt), chromatographic separation and Ion Ratio (IR) for identification in mass spectrometry was verified (SANTE/12089/2016). Quantification was carried out by external calibration in solvent.

### 4.6. Method Validation Procedure

For the method’s validation the following parameters were evaluated: LODs, LOQs, instrumental linearity ranges, recovery rates (%), RSDr and RSD_WLR_, both using relative standard deviation. All parameters’ definitions and acceptability criteria are reported in UNI CEN/TR 16059:2010.

#### 4.6.1. Limit of Detection and Limit of Quantification

LOD value were determined according to the “Estimation of LOD via blank samples” method as reported in the “Guidance Document on the Estimation of LOD and LOQ for Measurements in the Field of Contaminants in Feed and Food” [26]. Specifically, 10 aliquots of a blank matrix were spiked at 1 µg/kg for all EAs. These spiking levels were fixed as low as possible, considering a S/N ratio ≥3 at the expected LOD. The resulting spiked blank samples were analyzed by LC–MS/MS then an LOD and an LOQ were calculated according to Equations (1) and (2) respectively:

(1) $$\mathrm{LOD}=3.9*\frac{S_{y,b}}{b}$$

LOD: limit of detection

S_y,b_: standard deviation of the spiked blank signal

b: slope of calibration curve

LOQ = 3.3 ∗ LOD(2)

LOQ: limit of quantification

#### 4.6.2. Linearity Range

Each calibrant solution was prepared by diluting working solutions with acetonitrile/ammonium carbonate solution at 200 mg/L (50:50 *v*/*v*). Calibrant solutions were in the range 0.4–40 ng/mL and were analyzed on three different days over two weeks. Then calibration curve equations were obtained by plotting averaged peak areas vs. concentration of the natural toxin using ordinary least squares (OLS) method, including a (0, 0) point.

The linearity was checked as follows. For each calibration point, y-residuals were obtained by the following Equation (3)

(3) $$y-\mathrm{residuals}=y_{i}-\;{\hat{y}}_{i}$$

where $y_{i}$ are experimental values used for the regression equation calculation and ${\hat{y}}_{i}$ values are the points on the calculated regression line corresponding to individual x-values.

When, for all points, the residual along y axis were ≤±20%, the calibration curve was considered linear [17].

#### 4.6.3. Recovery, Repeatability and Within-Laboratory Reproducibility

Recoveries, RSDr and RSD_WLR_ for each molecule were evaluated according to UNI CEN/TR 16059:2010.

EAs validation was performed in wheat at three mass fraction levels, specifically 2.5, 5 and 10 μg kg^−1^ (corresponding to LOQ, 2xLOQ and 4xLOQ respectively) on two different days by two independent operators under repeatability conditions (eight replicates each). To obtain the WLR data, the two groups were combined and recovery% and RSD_WLR_ were calculated as reported in Table 2.

## Figures and Tables

**Table 1:** Maximum permitted level for ergot alkaloids in food by the European Commission (Commission Regulation (EU) 2021/1399). ^a^ Effective from 1 July 2024.

Foodstuff	Maximum Level for the Sum of 12 EAs µg/kg)
milling products of barley, wheat, spelt, oats grains(with an ash content lower than 900 mg/100 g)	100 (50 ^a^)
milling products of barley, wheat, spelt, oats (with an ash content equal or higher than 900 mg/100 g)	150
barley, wheat, spelt and oats grains placed on the market for the final consumer	150
rye milling products and rye placed on the market for the final consumer	500 (250 ^a^)
wheat gluten	400
processed cereal based food for infants and young children	20

**Table 2:** In-house analytical performances of the LC-MS/MS method for EAs, including spiking levels, limits of detection (LOD) and quantitation (LOQ), average recovery %, repeatability (RSDr) and within-laboratory reproducibility (RSD_WLR_). ^a^ Spiking levels were set at LOQ (2.5 µg/kg), 2xLOQ (5 µg/kg) and 4xLOQ (10 µg/kg).

	LOD (µg/kg)	LOQ (µg/kg)	Spiking Level ^a^ (µg/kg)	Mean Recovery, (%)	RSDr, (%)	RSD_WLR_, (%)
EM	0.3	0.8	2.5	97	6	7
			5	99	8	8
			10	108	8	8
EMI	0.2	0.6	2.5	111	7	11
			5	112	8	8
			10	119	5	5
ES	0.3	0.9	2.5	103	8	13
			5	101	9	13
			10	114	8	8
ESI	0.3	0.9	2.5	103	7	7
			5	110	7	8
			10	105	9	9
ET	0.3	1.1	2.5	105	8	8
			5	100	5	5
			10	105	10	11
ETI	0.2	0.7	2.5	111	8	10
			5	109	6	6
			10	113	4	4
EC	0.4	1.2	2.5	105	9	9
			5	95	13	13
			10	105	9	9
ECI	0.2	0.7	2.5	97	8	8
			5	97	11	11
			10	106	7	7
α EKR	0.7	2.1	2.5	105	8	10
			5	95	11	15
			10	104	7	9
α EKRI	0.2	0.8	2.5	87	12	12
			5	96	7	12
			10	100	8	8
ECR	0.7	2.3	2.5	105	10	10
			5	93	13	14
			10	108	13	14
ECRI	0.4	1.2	2.5	94	8	8
			5	99	8	8
			10	107	5	5

Abbreviation: ergometrine (EM), ergometrinine (EMI), ergosine (ES), ergosinine (ESI), ergotamine (ET), ergotaminine (ETI), ergocornine (EC), ergocorninine (ECI), α isomers of ergocryptine (α EKR), α isomers of ergocryptinine (α EKRI), ergocristine (ECR) and ergocristinine (ECRI).

**Table 3:** Concentration of EAs in cereal grains and cereal products (67 samples analysed). ^a^ Values calculated on positive samples. LC (left-censored data).

	Incidence	Mean ^a^ (µg/kg)	Range (µg/kg)	LC
EM	13	10.2	2.5–25	87
EMI	4	4.5	2.5–7.9	96
ES	10	7.4	2.5–23.5	90
ESI	4	4.7	2.5–6.2	96
ET	7	6.7	2.5–6.1	93
ETI	3	6.1	2.5–9.7	97
EC	6	8.8	2.5–13.9	94
ECI	3	7.5	2.5–12.4	97
EKR	7	9.5	2.5–27.8	93
EKRI	4	8.0	2.5–19.0	96
ECR	12	16.3	2.5–94.2	88
ECRI	7	12.4	2.5–48.3	93
Total EAs	25	31.2	2.7–270.7	75

Abbreviations: ergometrine (EM), ergometrinine (EMI), ergosine (ES), ergosinine (ESI), ergotamine (ET), ergotaminine (ETI), ergocornine (EC), ergocorninine (ECI), ergocryptine (EKR), ergocryptinine (EKRI), ergocristine (ECR) and ergocristinine (ECRI).

**Table 4:** Overview of representative studies on the occurrence of EAs in food samples collected worldwide over the period 2015–2021. The selected studies are relevant to data obtained from sets of more than 15 samples.

Country	Food Matrix	N Sample	Incidence %	Mean ^a^ (µg/kg)	Range (µg/kg)	References
Canada	barley	67	73	1150	2.2–29,425	[22]
Italy	rye-based products	16	7.5	NA	2.6–189	[18]
	wheat-based products	55	47	NA	2.5–1143	
China	cereal samples	123	4	204	9.5–803	[23]
Albania	cereals	228	NA	NA	65–1140	[24]
Algeria	barley	30	4	35.4	18–54	[25]
	wheat	30	8	33.1	3.7–76	
Belgium market	cereal based baby foods	49	49	3.1	0.1–41.6	[15]

N: number of analysed samples, ^a^ Values calculated on positive samples. NA: Not available in the publication.

**Table 5:** Retention times and monitored transitions for individual EAs.

ID	Retention Time (min)	Precursor Ion (*m*/*z*)	Product Ion (*m*/*z*)
EM	2.24	326	223
			208
EMI	2.82	326	208
			223
ES	4.60	548	223
			208
ESI	7.30	548	223
			208
ET	5.00	582	223
			208
ETI	7.95	582	223
			277
EC	5.85	562	223
			208
ECI	8.65	562	223
			277
α EKR	6.55	576	223
			268
α EKRI	9.50	576	223
			291
ECR	6.80	610	223
			268
ECRI	9.85	610	223
			208

Abbreviations: ergometrine (EM), ergometrinine (EMI), ergosine (ES), ergosinine (ESI), ergotamine (ET), ergotaminine (ETI), ergocornine (EC), ergocorninine (ECI), α isomers of ergocryptine (α EKR), α isomers of ergocryptinine (α EKRI), ergocristine (ECR) and ergocristinine (ECRI).

## Data Availability

The data presented in this study are available in Supplementary Material.

## Supplementary Materials

The following are available in online at https://www.mdpi.com/article/10.3390/toxins13120871/s1, Table S1: Occurrence data for Ergot Alkaloids: individual data for each toxin.
