# Supplementary material for: Undertaking a New Regulatory Challenge: Monitoring of Ergot Alkaloids in Italian Food Commodities
Source: Toxins (Basel). 2021 Dec 6;13(12):871. doi: 10.3390/toxins13120871 (PMC8708126; doi:10.3390/toxins13120871)
Supplement: Supplementary file 1 [file toxins-13-00871-s001.zip › toxins-1461420-supplementary.pdf]

Supplementary Materials: Undertaking a New Regulatory Challenge: Monitoring of Ergot Alkaloids in Italian Food Commodities

Veronica Maria Teresa Lattanzio, Emanuela Verdini, Stefano Sdogati, Angela Caporali, Biancamaria Ciasca and Ivan Pecorelli

Table S1. Occurrence data for Ergot Alkaloids: individual data for each toxin.

Region	Year	Samples	EM	EMI	ES	ESI	ET	ETI	EC	ECI	EKR	EKRI	ECR	ECRI	Sum
Puglia	2017	Wheat flour	N.D.	N.D.	N.D.	N.D.	N.D.	N.D.	N.D.	N.D.	N.D.	N.D.	N.D.	N.D.	N.D.
Umbria	2017	Barley grain	N.D.	N.D.	N.D.	N.D.	N.D.	N.D.	N.D.	N.D.	N.D.	N.D.	N.D.	N.D.	N.D.
Umbria	2017	Corn grain	N.D.	N.D.	N.D.	N.D.	N.D.	N.D.	N.D.	N.D.	N.D.	N.D.	N.D.	N.D.	N.D.
Marche	2017	Spelt grain	N.D.	N.D.	N.D.	N.D.	N.D.	N.D.	N.D.	N.D.	N.D.	N.D.	N.D.	N.D.	N.D.
Umbria	2017	Spelt grain	N.D.	N.D.	N.D.	N.D.	N.D.	N.D.	N.D.	N.D.	N.D.	N.D.	N.D.	N.D.	N.D.
Umbria	2017	Wheat grain	N.D.	N.D.	N.D.	N.D.	N.D.	N.D.	N.D.	N.D.	N.D.	N.D.	N.D.	N.D.	N.D.
Umbria	2017	Wheat grain	N.D.	N.D.	N.D.	N.D.	N.D.	N.D.	N.D.	N.D.	N.D.	N.D.	N.D.	N.D.	N.D.
Umbria	2017	Wheat grain	6.2	N.D.	N.D.	N.D.	N.D.	N.D.	N.D.	N.D.	N.D.	N.D.	N.D.	N.D.	6.2
Marche	2017	Wheat grain	9.3	7.9	5.7	N.D.	N.D.	N.D.	N.D.	N.D.	N.D.	N.D.	N.D.	N.D.	23
Umbria	2017	Bakery products	5.5	N.D.	3.8	N.D.	N.D.	N.D.	N.D.	N.D.	N.D.	N.D.	N.D.	N.D.	9.3
Marche	2017	Bread	N.D.	N.D.	N.D.	N.D.	N.D.	N.D.	N.D.	N.D.	N.D.	N.D.	N.D.	N.D.	N.D.
Umbria	2017	Cereal flakes	20	N.D.	N.D.	N.D.	N.D.	N.D.	N.D.	N.D.	N.D.	N.D.	N.D.	N.D.	20
Umbria	2017	Crackers	N.D.	N.D.	N.D.	N.D.	N.D.	N.D.	N.D.	N.D.	5.6	N.D.	N.D.	N.D.	5.6
Marche	2017	Pasta	5.8	N.D.	N.D.	N.D.	6.1	N.D.	N.D.	N.D.	3.6	2.6	7.9	3.8	29.8
Umbria	2017	Pasta	N.D.	N.D.	4.1	N.D.	N.D.	N.D.	N.D.	N.D.	N.D.	N.D.	N.D.	N.D.	4.1
Umbria	2018	Wheat bran	9.9	3.1	9.0	5.4	18	9.7	14	12	28	19	94	48	271
Marche	2018	Wheat flour	N.D.	N.D.	N.D.	N.D.	N.D.	N.D.	N.D.	N.D.	N.D.	N.D.	N.D.	N.D.	N.D.
Marche	2018	Wheat flour	N.D.	N.D.	N.D.	N.D.	N.D.	N.D.	N.D.	N.D.	N.D.	N.D.	N.D.	N.D.	N.D.
Puglia	2018	Wheat flour	N.D.	N.D.	N.D.	N.D.	N.D.	N.D.	N.D.	N.D.	N.D.	N.D.	2.7	N.D.	2.7
Umbria	2018	Barley grain	N.D.	N.D.	N.D.	N.D.	N.D.	N.D.	N.D.	N.D.	N.D.	N.D.	N.D.	N.D.	N.D.
Umbria	2018	Corn grain	N.D.	N.D.	N.D.	N.D.	N.D.	N.D.	N.D.	N.D.	N.D.	N.D.	N.D.	N.D.	N.D.
Umbria	2018	Oats grain	N.D.	N.D.	N.D.	N.D.	N.D.	N.D.	N.D.	N.D.	N.D.	N.D.	N.D.	N.D.	N.D.
Umbria	2018	Spelt grain	N.D.	N.D.	N.D.	N.D.	N.D.	N.D.	N.D.	N.D.	N.D.	N.D.	N.D.	N.D.	N.D.
Umbria	2018	Spelt grain	N.D.	N.D.	N.D.	N.D.	N.D.	N.D.	N.D.	N.D.	N.D.	N.D.	N.D.	N.D.	N.D.
Umbria	2018	Spelt grain	N.D.	N.D.	N.D.	N.D.	N.D.	N.D.	N.D.	N.D.	N.D.	N.D.	N.D.	N.D.	N.D.
Marche	2018	Wheat grain	N.D.	N.D.	N.D.	N.D.	N.D.	N.D.	N.D.	N.D.	N.D.	N.D.	N.D.	N.D.	N.D.
Puglia	2018	Wheat grain	25	N.D.	24	6.2	N.D.	N.D.	11	N.D.	7.8	N.D.	N.D.	3.1	77
Umbria	2018	Biscuits	N.D.	N.D.	N.D.	N.D.	N.D.	N.D.	N.D.	N.D.	N.D.	N.D.	N.D.	N.D.	N.D.
Marche	2018	Bread	N.D.	N.D.	N.D.	N.D.	3.9	N.D.	N.D.	N.D.	N.D.	N.D.	2.6	N.D.	6.5
Umbria	2018	Cereal flakes	7.6	N.D.	N.D.	N.D.	N.D.	N.D.	7.6	N.D.	N.D.	N.D.	N.D.	N.D.	15
Umbria	2018	Cereal flakes	N.D.	N.D.	N.D.	N.D.	N.D.	N.D.	N.D.	N.D.	N.D.	N.D.	N.D.	N.D.	N.D.
Umbria	2018	Cereal flakes	N.D.	N.D.	N.D.	N.D.	N.D.	N.D.	N.D.	N.D.	N.D.	N.D.	N.D.	N.D.	N.D.
Marche	2019	Spelled flour	N.D.	N.D.	N.D.	N.D.	N.D.	N.D.	N.D.	N.D.	N.D.	N.D.	N.D.	N.D.	N.D.
Umbria	2019	Spelled flour	N.D.	N.D.	N.D.	N.D.	N.D.	N.D.	N.D.	N.D.	N.D.	N.D.	N.D.	N.D.	N.D.
Marche	2019	Wheat flour	N.D.	N.D.	N.D.	N.D.	N.D.	N.D.	N.D.	N.D.	N.D.	N.D.	N.D.	N.D.	N.D.
Marche	2019	Wheat flour	N.D.	N.D.	N.D.	N.D.	N.D.	N.D.	N.D.	N.D.	N.D.	N.D.	3.4	N.D.	3.4

Umbria2019	Wheat flour	N.D.	N.D.	2.9	N.D.	3.0	N.D.	N.D.	N.D.	N.D.	N.D.	6.0	N.D.	12
Marche2019	Wheat flour	N.D.	N.D.	N.D.	N.D.	N.D.	N.D.	N.D.	N.D.	N.D.	N.D.	11	4.1	15
Umbria2019	Wheat flour	N.D.	N.D.	N.D.	N.D.	N.D.	N.D.	N.D.	N.D.	N.D.	N.D.	N.D.	N.D.	N.D.
Marche2019	Wheat flour	N.D.	N.D.	N.D.	N.D.	N.D.	N.D.	N.D.	N.D.	N.D.	N.D.	N.D.	N.D.	N.D.
Marche2019	Wheat flour	N.D.	N.D.	N.D.	N.D.	N.D.	N.D.	N.D.	N.D.	N.D.	N.D.	N.D.	N.D.	N.D.
Marche2019	Wheat flour	N.D.	N.D.	N.D.	N.D.	N.D.	N.D.	N.D.	N.D.	N.D.	N.D.	N.D.	N.D.	N.D.
Marche2019	Oats grain	N.D.	N.D.	N.D.	N.D.	N.D.	N.D.	N.D.	N.D.	N.D.	N.D.	N.D.	N.D.	N.D.
Umbria2019	Wheat grain	N.D.	N.D.	N.D.	N.D.	N.D.	N.D.	N.D.	N.D.	N.D.	N.D.	N.D.	N.D.	N.D.
Marche2019	Biscuits for infants	N.D.	N.D.	N.D.	N.D.	N.D.	N.D.	N.D.	N.D.	N.D.	N.D.	N.D.	N.D.	N.D.
Umbria2019	Biscuits for infants	N.D.	N.D.	N.D.	N.D.	N.D.	N.D.	N.D.	N.D.	N.D.	N.D.	N.D.	N.D.	N.D.
Marche2019	Cereal-based food for infants	N.D.	N.D.	N.D.	N.D.	N.D.	N.D.	N.D.	N.D.	N.D.	N.D.	N.D.	N.D.	N.D.
Marche2019	Pasta for infants	N.D.	N.D.	N.D.	N.D.	N.D.	N.D.	N.D.	N.D.	N.D.	N.D.	N.D.	N.D.	N.D.
Marche2019	Pasta for infants	N.D.	N.D.	N.D.	N.D.	N.D.	N.D.	N.D.	N.D.	N.D.	N.D.	N.D.	N.D.	N.D.
Marche2019	Pasta	N.D.	N.D.	N.D.	N.D.	N.D.	N.D.	N.D.	N.D.	N.D.	N.D.	N.D.	N.D.	N.D.
Marche2019	Bakery products	N.D.	N.D.	N.D.	N.D.	N.D.	N.D.	N.D.	N.D.	N.D.	N.D.	N.D.	N.D.	N.D.
Umbria2019	Bakery products	N.D.	N.D.	N.D.	N.D.	N.D.	N.D.	N.D.	N.D.	N.D.	N.D.	N.D.	N.D.	N.D.
Marche2019	Biscuits	N.D.	N.D.	N.D.	N.D.	N.D.	N.D.	N.D.	N.D.	N.D.	N.D.	N.D.	N.D.	N.D.
Umbria2019	Biscuits	N.D.	N.D.	N.D.	N.D.	N.D.	N.D.	N.D.	N.D.	N.D.	N.D.	N.D.	N.D.	N.D.
Umbria2019	Cereal flakes	N.D.	N.D.	N.D.	N.D.	N.D.	N.D.	N.D.	N.D.	N.D.	N.D.	N.D.	N.D.	N.D.
Umbria2020	Wheat flour	2.5	N.D.	N.D.	N.D.	2.5	2.5	N.D.	N.D.	2.5	2.5	2.5	2.5	18
Umbria2020	Wheat flour	N.D.	2.5	2.5	2.5	N.D.	N.D.	2.5	2.5	N.D.	N.D.	N.D.	N.D.	13
Umbria2020	Cereal-based food for infants	N.D.	N.D.	N.D.	N.D.	N.D.	N.D.	N.D.	N.D.	N.D.	N.D.	N.D.	N.D.	N.D.
Umbria2020	Cereal-based food for infants	N.D.	N.D.	N.D.	N.D.	N.D.	N.D.	N.D.	N.D.	N.D.	N.D.	N.D.	N.D.	N.D.
Marche2020	Cereal-based food for infants	N.D.	N.D.	N.D.	N.D.	N.D.	N.D.	N.D.	N.D.	N.D.	N.D.	N.D.	N.D.	N.D.
Umbria2020	Pasta for infants	N.D.	N.D.	N.D.	N.D.	N.D.	N.D.	N.D.	N.D.	N.D.	N.D.	N.D.	N.D.	N.D.
Puglia 2020	Multicereal flakes	N.D.	N.D.	N.D.	N.D.	N.D.	N.D.	N.D.	N.D.	N.D.	N.D.	N.D.	N.D.	N.D.
Marche2020	Multicereal flakes	N.D.	N.D.	N.D.	N.D.	N.D.	N.D.	N.D.	N.D.	N.D.	N.D.	N.D.	N.D.	N.D.
Marche2020	Multicereal flakes	N.D.	N.D.	N.D.	N.D.	N.D.	N.D.	N.D.	N.D.	N.D.	N.D.	N.D.	N.D.	N.D.
Umbria2020	Multicereal flakes	N.D.	N.D.	N.D.	N.D.	N.D.	N.D.	N.D.	N.D.	N.D.	N.D.	N.D.	N.D.	N.D.
Umbria2020	Multigrain bread	N.D.	N.D.	N.D.	N.D.	N.D.	N.D.	N.D.	N.D.	N.D.	N.D.	N.D.	N.D.	N.D.
Umbria2020	Rusks	N.D.	N.D.	N.D.	N.D.	N.D.	N.D.	N.D.	N.D.	N.D.	N.D.	N.D.	N.D.	N.D.

ergometrine (EM), ergometrinine (EMI), ergosine (ES), ergosinine (ESI), ergotamine (ET), ergotaminine (ETI), ergocornine (EC), ergocorninine (ECI), mixture of α- and β-isomers of ergocryptine (EKR) and ergocryptinine (EKRI), ergocristine (ECR) and ergocristinine (ECRI) .
